# Characterization of inhibitory T cells induced by an analog of type II collagen in an HLA-DR1 humanized mouse model of autoimmune arthritis

**DOI:** 10.1186/ar3832

**Published:** 2012-05-08

**Authors:** Masaru Kimata, David L Cullins, Monica L Brown, David D Brand, Edward F Rosloniec, Linda K Myers, John M Stuart, Andrew H Kang

**Affiliations:** 1 Department of Medicine, University of Tennessee Health Science Center, 956 Court Ave., Room G326, Memphis TN, 38163, USA; 2 Department of Pediatrics, University of Tennessee Health Science Center, 956 Court Ave., Room G326, Memphis TN 38163, USA; 3 Research Service, Veterans Affairs Medical Center, 1030 Jefferson Ave, Memphis TN 38104, USA

## Abstract

**Introduction:**

We used DR1 transgenic mice and covalently linked DR1 multimers to characterize analog-specific inhibitory T cells in collagen-induced arthritis (CIA). Because of the low numbers of antigen-specific T cells in wild-type mice, functional T-cell studies in autoimmune arthritis have been challenging. The use of T-cell receptor (TCR) transgenic mice has provided useful information, but such T cells may not represent the heterogeneous T-cell response that occurs in natural settings. Our focus was to develop tools to identify and characterize the population of immunoregulatory T cells induced in wild-type mice by an analog peptide of CII_259-273_, which contains amino acid substitutions at positions 263 (N) and 266 (D) (analog peptide A12).

**Methods:**

DR1 multimers, developed by loading empty class II molecules with exogenous peptide, provide a method for visualizing antigen-specific T cells with flow cytometry. However, the low binding avidity of A12 for the major histocompatibility complex (MHC) made this strategy untenable. To overcome this problem, we generated DR1 multimers in which the analog peptide A12 was covalently linked, hoping that the low-avidity analog would occupy enough binding clefts to allow detection of the responsive T cells.

**Results:**

Staining with the tetramer revealed that A12-specific T cells were readily detectable at 10 days after immunization. These CD4(+) T cells are a highly selective subset of the TCR repertoire and have a limited clonality. Analysis of cytokine expression showed that cells detected by tetramer (A12) expressed primarily suppressive cytokines (interleukin-4 (IL-4) and IL-10) in response to collagen, compared with control cells. Although they did not express Fox-p3, they were extremely effective in preventing and suppressing inflammatory arthritis.

**Conclusions:**

In summary, our studies showed that the use of covalently linked multimers allows characterization of analog-specific T cells that are otherwise difficult to detect. The suppressive character of the analog-specific T-cell response suggests that these cells attenuate autoimmunity and differ significantly in phenotype from the inflammatory T cells predominantly found in arthritic joints. Such reagents will become powerful tools to study T-cell responses in RA patients in upcoming clinical trials.

## Introduction

Rheumatoid arthritis (RA) is a systemic disease consisting of chronic inflammation in multiple articular joints. Current treatments such as systemic anti-tumor necrosis factor (TNF)-α therapies are effective in > 50% of RA patients [1]; however, biologics must be given in relatively high dosages, and significant side effects have been reported [2]. The success of the best-known altered peptide ligand, Copaxone (Teva Pharmaceuticals, Petach Tikva, Israel.) as a first-line therapy for multiple sclerosis (MS), suggests that peptide therapies based on naturally occurring proteins may provide an effective alternative to drug biologics for the treatment of arthritis. Although various strategies have been tested, we have developed a unique modification to a protein that is the predominant collagen type found in human cartilage. Our therapy is based on our previous discovery of an analog peptide of type II collagen (CII) that could profoundly suppress arthritis in the CIA model by inducing a unique inhibitory T cell [3].

Although the original observations were made by using DBA/1 mice, we have extended our findings to a humanized animal model specifically developed to mimic more closely the pathogenic features of RA. In this model, mice transgenic for one of the most common RA-susceptible major histocompatibility complex (MHC) molecules, DRB*0101 (DR1) [4,5] become arthritic when immunized with CII/CFA. By using proliferation and cytokine assays, we determined that a peptide representing CII_263-270 _and containing amino acid substitutions at positions 263 (F→N) and 266 (E→D) (analog peptide A12) was profoundly suppressive, effectively attenuating arthritis in the humanized RA-mouse models, despite having very poor avidity for the MHC [6].

Although collagen peptides designed with carefully crafted substitutions such as A12 may provide an attractive alternative choice for treatment of RA, the low numbers of antigen-specific T cells in wild-type mice have made functional T-cell studies in autoimmune arthritis particularly challenging. Studies using TCR transgenic T cells are limited to the study of one TCR, and the low MHC binding avidity of peptide A12 makes untenable the strategy of loading empty class II molecules with exogenous peptide to be assembled as MHC multimers (tetramers). To overcome this problem, we generated DR1 multimers [7,8], in which the analog peptide A12 was covalently linked, making it more likely that majority of the MHC molecule binding clefts are occupied by this peptide [9-11]. By using the tetramers as a tool to study analog-responsive T cells, we immunized DR1 tg mice with A12/CFA and isolated draining inguinal lymph node cells. Culture with the tetramer revealed that A12-specific T cells were readily detectable. Moreover, the A12-responsive T cells could be visualized to characterize the TCR-Vβ repertoire and cytokine secretion profiles. Adoptive transfer experiments demonstrated that tetramer^+ ^cells were very effective in preventing and suppressing inflammatory arthritis.

Looking to the future, class II tetramers, such as DR1-A12 show promise for monitoring the development of analog-specific T cells in clinical settings whenever low-avidity analog peptides will be used to treat patients with RA.

## Materials and methods

### Preparation of tissue-derived CII and synthetic peptides

Native CII was solubilized from fetal calf articular cartilage by limited pepsin-digestion and purified as described earlier [12]. The purified collagen was dissolved in cold 20 m*M *acetic acid at 4 mg/ml and stored frozen at -70°C until used. The synthetic peptides were supplied by Biomolecules Midwest Inc. (Waterloo, IL, USA). A peptide containing the immunodominant determinant sequence of CII (GIAGFKGEQGPKGEB) is referred to as A2 or wild-type (WT) IEDBID 109115; a synthetic peptide representing the sequence (GIAGNKGDQGPKGEB) is designated A12.

### Animals

#### Generation of Tg mice expressing DR

Mice expressing the chimeric (human/mouse) DRB1*0101 construct were maintained in our onsite pathogen-free facility. The chimeric DRB1*0101 construct has been previously described, as has the production of Tg mice expressing these constructs [4].

#### Generation of DR1 mice that are IL4 or IL10 knockout

The IL-4 (C57BL/6-^*IL4tm1cgn *^IL-4 and C57BL/6-^*IL10tm1cgn *^knockout) mice were purchased from Jackson Laboratory (Bar Harbor, ME, USA) and backcrossed onto the arthritis-susceptible DR1 C57BL/10 background for 12 generations before they were intercrossed. Genomic DNA was obtained from blood samples, and polymerase chain reaction (PCR) was used to identify mice homozygous for the IL-4^-/- ^and IL-10^-/- ^phenotypes.

All mice were fed standard rodent chow (Ralston Purina Co., St. Louis, MO, USA) and water *ad libitum*. To ensure that the environment was pathogen free, sentinel mice were routinely tested for hepatitis and Sendai viruses. All animals were kept until the age of 7 to 10 weeks before being used for experiments, which were conducted in accordance with approved IACUC protocols.

### Immunization

CII was dissolved in 10 m*M *acetic acid at a concentration of 4 mg/ml and emulsified with an equal volume of CFA containing 4 mg/ml of *Mycobacterium tuberculosis *strain H37 Ra (Difco Microbiology Products, Becton Dickinson, Franklin Lakes, NJ, USA) [12]. For the induction of arthritis, each mouse received 100 μg of CII emulsified in CFA subcutaneously at the base of the tail. For other immunizations, each mouse received subcutaneously 100 μg of either peptide A12 or Ova emulsified with CFA.

### Measurement of the severity of arthritis

The severity of arthritis were determined by visually examining each forepaw and hindpaw and scoring them on a scale of 0 to 4, as described previously [12]. Scoring was conducted by two examiners, one of whom was unaware of the identity of the treatment groups. Each mouse was scored thrice weekly, beginning 3 weeks after immunization and continuing for 8 weeks. The mean severity score (sum of the severity scores for the group on each day/total number of animals in the group) was recorded at each time point.

### Production of soluble HLA-DR protein

Soluble DR1 was purified from culture supernatants of S2 *Drosophila *cells previously transfected with DRB1*0101 and DRA1*0101. Both the α- and β-chains of the DR1 constructs contain murine I-E leader sequences, followed by DRα or DRβ first domains and murine I-Eα and I-Eβ second domains. The analog (CII259-273, 263F, 266D), was inserted after the third residue of the β-chain, and a flexible ((Gly)4-Ser)3 linker was added to allow the peptide to fold into the binding groove of the DR molecule. The sequences encoding the cytoplasmic and transmembrane portions of the molecules were deleted from the cDNA by PCR, and a new stop codon was inserted at the 3' end of the second domain of the α-chain. The β-chains were altered by adding a linker and a biotinylation site 3' to the Eβ second domain, to allow site-specific addition of biotin and tetramerization by using streptavidin. The resulting cDNA was cloned into the *Drosophila *expression vector pRmHA-3 (gift from Dr. D. Zaller, Merck, Rahway, NJ, USA). S2 cells were transfected with a 10:10:1 ratio of chimeric DRB1 and DRA1 to pUChsNeo by using calcium phosphate precipitation. Soluble DR production was induced by 1 m*M *CuSO4, and 5 days later, the culture supernatant was collected and adjusted to 0.05% octyl glucoside (OcG). The DR molecules were purified by passage of the culture supernatant over an affinity column coupled with the anti-DR Ab LB3.1. The column was washed with 0.05% OcG and 0.15 *M *NaCl in phosphate buffer, pH 7.5, followed by 0.05% OcG and 0.5 *M *NaCl in phosphate buffer, pH 7.5, and a final wash with 10 m*M *Tris in 0.5 *M *NaCl, pH 7.5. The DR molecules were eluted with 100 m*M *Tris and 0.5 *M *NaCl, pH 11.2, and the fractions were immediately neutralized with acetic acid. The DR recovered was concentrated by using an Amicon Stirred Cell, quantitated by OD280 absorption and ELISA (PathScan Total DDR1 Sandwich ELISA Kit; Cell Signaling, Danvers, MA, USA), and was evaluated with SDS-PAGE before use. Biotinylation of the proteins was performed with BirA (Avidity, Denver, CO, USA) and incorporated into saturated complexes with phycoerythrin (PE)-streptavidin (BioSource International, Camarillo, CA, USA) before use.

### Tetramer binding

For tetramer staining of T cells, lymph node cells were diluted to a concentration of 2.5 × 10^7^/ml in DMEM medium (Gibco) supplemented with 50 U/ml penicillin G sodium, 50 μg/ml streptomycin sulfate, 0.05 m*M *2-ME, 2 m*M *L-glutamine, and 0.1% BSA, and T-hybridoma cells were diluted to a concentration of 2.5 × 10^6^/ml in DMEM complete. One million lymph node cells or 10^5 ^T-hybridoma cells were then aliquoted into a 96-well plate and were incubated with 1 μg of tetramer in 10 μl of complete medium supplemented with 5 m*M *NaN_3_. Cells were incubated at 37°C for 2.5 hours, at which time, antibodies to cell-surface markers were added, and cells were incubated for an additional 30 minutes at 4°C. After incubation with Abs to cell-surface markers, samples were washed 3 times with 200 μl of PBS supplemented with 0.1% NaN_3 _and 2% FBS, resuspended in 200 μl of PBS supplemented with 0.1% NaN_3 _and 2% FBS, and analyzed with flow cytometry by using a FACSCalibur (BD Biosciences). In some experiments, the draining lymph nodes of DR1 mice were harvested 10 days after immunization with CII and were incubated with tetramer and subjected to magnetic sorting to obtain a purified population of tetramer-positive cells (Miltenyi Biotec).

### Flow cytometry analysis of TCR repertoire

To determine the frequency of individual Vβ subfamilies expressed by tetramer^+ ^T cells in DR1 mice, draining inguinal cells from DR1 mice immunized 10 days earlier with A12/CFA were incubated with tetramer labeled with PE, anti-CD4-APC, anti-α/βTCR-PerCP (BD Biosciences, San Jose, CA, USA), and an anti-TCR-Vβ-specific Ab conjugated with FITC for 30 minutes at 4°C (Mouse Vβ TCR Screening Panel; BD Biosciences). Labeled cells were washed with PBS, and a minimum of 10,000 cells was analyzed from each sample with flow cytometry by using a FACSCalibur or LSR II (BD Biosciences). The final analysis was performed by using FlowJo software (Tree Star, Ashland, OR, USA).

### Measurement of serum antibody titers

Mice were bled at 6 weeks after immunization, and sera were analyzed for antibodies reactive with native CII by using a modification of an enzyme-linked immunoassay (ELISA) previously described [12]. Serial dilutions of a standard serum were added to each plate. From these values, a standard curve was derived by computer analysis by using a four-parameter logistic curve. Results are reported as units of activity, derived by comparison of test sera with the curve derived from the standard serum, which was arbitrarily defined as having 50 units of activity. Reactivity to CII was not detected in sera obtained from normal mice.

### Phenotypic characterization of the cells

Inguinal lymph nodes from immunized mice were isolated, and the phenotype induced by A12 was determined by multiparameter flow cytometry with an LSRII flow cytometer (BD Biosciences). Cells were cultured with fluorochrome-labeled antibodies specific for CD4, CD25, or CD44 (BD Biosciences) as well as tetramer. In some experiments, intracellular labeling was performed by using antibodies specific for FoxP3 (FJK-16s; eBioscience, San Diego, CA, USA), per the manufacturer's protocol. In the experiments involving intracellular staining, specific gating was performed on both the CD4^+ ^and the Vβ8^+^, Vβ14^+ ^population of cells.

### Passive-transfer experiments

In transfer experiments, inguinal lymph node cells from DR1 mice previously immunized with the A12 analog peptide in CFA were collected 8 days after immunization, and various cell subsets were fractionated by using PE-labeled tetramers and ferromagnetic beads (Miltenyi Biotec, Gladbach, Germany), according to the manufacturer's protocol. The purity of each cell population was confirmed by flow cytometry to have > 95% purity. Recipient mice were given cells intravenously by using two protocols (a) 2 × 10^5 ^either tetramer+ or tetramer^- ^T cell or (b) 5 × 10^5 ^of VB8^+^, VB14^+ ^T cells. All recipient mice were immunized with CII either on the day of the cell transfer (prevention protocol) or 25 days before cell transfer (therapy protocol) and observed for arthritis.

### Measurement of cytokines

To measure cytokines, inguinal CD4^+ ^lymph node cells or purified tetramer^+ ^T cells were cultured (5 × 10^5 ^CD4^+ ^T cells/ml) with wild-type APCs (DR1-positive splenocytes) (1:2 ratio), which had been prepulsed with 100 μg/ml of the various peptides (A2 or A12). The CD4^+ ^cells were collected 8 days after immunization (with either OVA, CII, or A12 emulsified in CFA) by negative selection by using ferromagnetic beads, according to the manufacturer's protocol (Miltinyi Biotech). (Each cell population was confirmed by flow cytometry to have > 95% purity). Each culture was set up by using pooled cells from three mice run in triplicates, and supernatants were collected at 72 hours and analyzed for the presence of IL-4, IL-10, IL-2, INF-γ, and IL-17 by using a Bio-plex mouse cytokine assay (Bio-Rad, Hercules, CA, USA) according to the manufacturer's protocol. Values are expressed as picograms per milliliter and represent the mean values for each group taken from three separate experiments.

### Statistical analysis

Mean severity scores, antibody titers, and cytokine levels were compared by using the Mann-Whitney test.

## Results

### Functional analysis of chimeric recombinant soluble DR molecules

**Figure 1:**
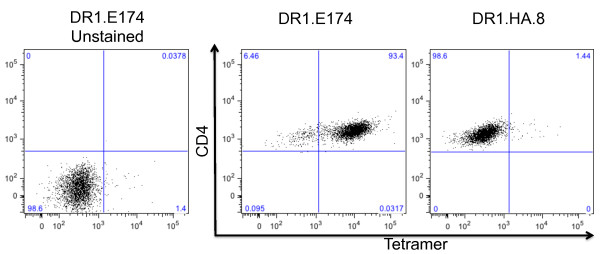
**Functional analysis and specificity of the recombinant DR1-A12**. Tetrameric DR1-A12 was tested for its ability to bind specifically to DR1-restricted, CII-specific T cells. A panel of T-cell hybridomas was cultured with the tetramers in saturating concentrations at 37°C for 3 hours. An anti-CD4 antibody was added in the last 30 minutes before the cells were washed and analyzed on the flow cytometer. The left panel demonstrates an unstained control. The middle panel shows hybridoma DR1.E174, which is representative of tests performed on nine individual CII-specific DR1-restricted hybridomas. The right panel (DR1.HA.8) is representative of four control T-cell hybridomas specific for an HA peptide in the context of DR1. Previous testing has shown that none of these hybridomas is bound by the irrelevant tetramer DR1-HA, which contains the immunodominant determinant of hemagglutinin [8]). These data confirm that that our A12/DR1-tetramer recognizes only the collagen-specific, DR1-restricted T cells in our panel.

We previously reported that the A12-induced suppression of arthritis is achieved primarily by T cells [13]. To identify and characterize clearly the cells responsible for this effect, we generated DR1 multimers (tetramers) that have a covalently linked collagen analog, so that the A12 peptide occupies the majority of the MHC molecule binding clefts [9-11]. Biotinylated DR1-A12 molecules were generated and assembled into tetramers by using phycoerythrin-streptavidin. To determine the binding specificity of our tetramer, we used a well-characterized panel of DR1-restricted CII-specific T-cell hybridomas [8,14] and cultured them with the A12-tetramer before analysis with flow cytometry. As shown in Figure 1, the A12-tetramer bound only to the collagen-specific DR1 restricted hybridomas (example, DR1-E174, middle panel, Figure 1, which is representative of nine individual CII-specific hybridomas (mean fluorescence (MF) = 11,400 ± 392). Conversely, the tetramer was antigen specific, unable to recognize DR1-restricted hybridomas that are specific for hemagglutinin (example, DR1.HA.8; right panel, Figure 1, a DR-1 restricted hybridoma that recognized HA, MF = 493 ± 256; *n *= 4). Finally, we determined that the tetramer binding was MHC specific, incapable of binding collagen-specific hybridomas that were restricted by another MHC (for example, DR4-restricted collagen-specific hybridomas, MF = 462 ± 280, *n *= 4, *P *< 0.0001; compared with the MF of 11,400 ± 392 of the DR1-restricted CII-specific hybridomas, *n *= 9). These data confirm that that our A12/DR1-tetramer recognizes only the collagen-specific, DR1-restricted T cells in our panel.

### Identification of A12-specific T cells *in vivo*

**Figure 2:**
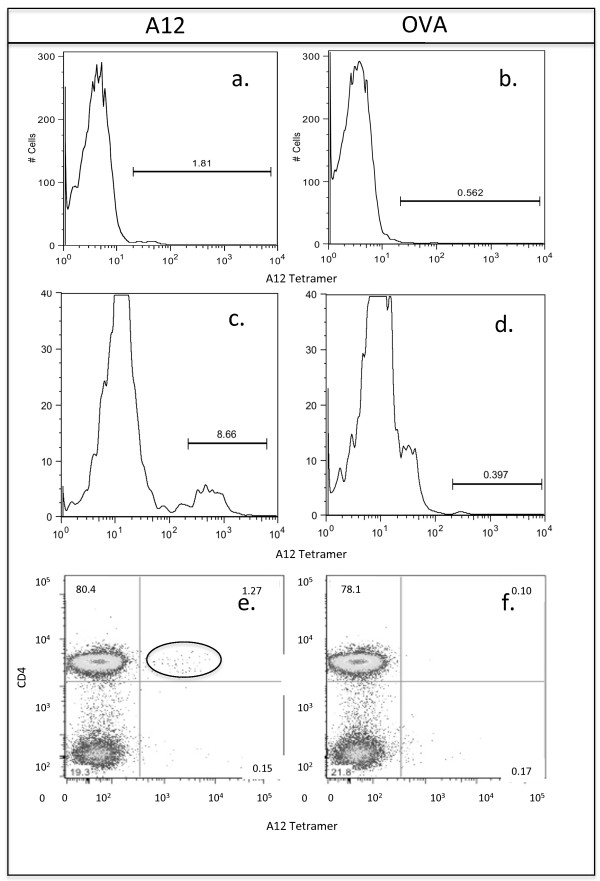
***Ex vivo *analysis of the development of the A12-specific T-cell response in DR1 Tg mice**. DR1 mice were immunized with either A12 or OVA, and individual mice were killed on day 10 after immunization. Cells from inguinal lymph nodes were collected and either analyzed immediately **(a, b) **or stimulated for 4 days with the A12 peptide or Ova before analysis **(c, d)**. (Lower panel) DR1 mice were immunized with either CII/CFA **(e) **or Ova **(f) **and, at the time of onset of arthritis, were treated subcutaneously (100 μg/mouse in IFA) with either A12 peptide **(e) **or Ova (right) **(f)**. Cells were collected from inguinal lymph nodes on day 26 after treatment with peptide, and the cells were analyzed for the presence of tetramer-positive cells directly *ex vivo *with flow cytometry. All cells were analyzed for the presence of A12-specific T cells by using the DR1-A12 tetramer and anti-CD3 or anti-CD4, anti-CD8, and anti-CD19. Anti-CD8 and anti-CD19 were used simultaneously in the FL4 channel, and anti-CD3 or anti-CD4 was used in the FL1 channel. The cells represented are CD19^-^/CD8^-^/CD3^+ ^or CD19^-^/CD8^-^/CD4^+^. Data are representative of at least six mice per time point and are based on a minimum of 100,000 cells analyzed.

Although the concept of "suppressor" T cells was first put forward 40 years ago [15], an increasing number of immune cell populations have been reported to play a role in the regulation of autoimmunity [16]. Therefore, it is important to characterize precisely the cells induced by the analog peptide A12. We chose to search for the A12-specific T cells directly *ex vivo*. DR1 mice were immunized with A12 or Ova (as a control), and 10 days later, T cells from inguinal lymph nodes were stained with DR1-A12 tetramer and anti-CD3, either immediately or after 4 days of restimulation in culture with the A12 peptide. As shown in Figure 2, an increase in the percentage and number of CD3^+ ^T cells that bound the DR1-A12 tetramer could be observed both *ex vivo *and after stimulation in culture compared with controls. Although the *ex vivo *analyses of DR1-restricted, A12-specific T cells revealed only a small population of tetramer^+^/CD3^+ ^T cells (1.5% ± 0.3% versus 0.5% ± 0.3%; *P *≤ 0.05; *n *= 6), by day 4 of stimulation in culture, a clearly discernible population (8.5% ± 0.5% versus 0.5% ± 0.4%; *P *≤ 0.05; *n *= 6), representing a fivefold expansion of cells, was visible. The specificity of the tetramer staining was quite clear, because nonspecific binding (cells derived from DR1 mice that had been immunized with Ova, but stained with a DR1/A12 peptide tetramer) was extremely low (Figure 2b, d).

In clinical settings, analog peptides will be administered therapeutically during episodes of active arthritis. To characterize the A12-specific T-cell response that develops within an inflammatory milieu, DR1 mice were immunized with CII/CFA, and once arthritis was clearly established, the mice were treated subcutaneously with A12 peptide. When the DR1-A12 multimers were used to stain inguinal lymph node cells from arthritic mice evaluated 26 days after peptide treatment, a distinct population of tetramer^+ ^cells was detected in the A12-treated mice (1.2% ± 0.5% versus 0.2% ± 0.3%; *P *≤ 0.05; *n *= 6; Figure 2e). Again the specificity of the tetramer staining was clear, because nonspecific tetramer binding by cells derived from DR1 mice that had been immunized with Ova remained low (Figure 2f).

### Oligoclonality and phenotype of A12-specific T cells

Although we previously reported that CII-specific T cells restricted by DR1 are clearly oligoclonal [8], the degree of heterogeneity among analog A12-responding T cells remained unclear. Therefore, we analyzed the T cells that migrated to the inguinal lymph nodes after immunization with A12 and analyzed them for TCR β-chain variability. As shown in Table 1, when cells were analyzed by specifically gating on individual Vβ populations after co-staining with tetramer, two Vβ types were explicitly preferred, Vβ8 (Vβ8.1, Vβ8.2, or Vβ8.3) and Vβ14. A significant tetramer^+ ^T-cell population was detected in each of these two subsets (Vβ8 and Vβ14), whereas all other Vβ antibodies tested had tetramer levels indistinguishable from background. Collectively, Vβ14 and Vβ8 accounted for > 97% of the tetramer^+ ^T cells, thus identifying them as the major T-cell population responding to the A12 analog determinant presented by DR1. The tetramer-negative cells had a T-cell repertoire comparable to that of wild-type DR1 mice, which we previously reported [8].

**Table 1:** Analysis of TCR Vβ use in A12-tetramer^+ ^T cells

TCR Vβ	% Tetramer^+^
VB2	< 1

VB3	< 1

VB4	< 1

VB5 (5.1, 5.2).	< 1

VB6	< 1

VB7	< 1

VB8 (8.1, 8.2, 8.3)	29 ± 15

VB9	< 1

VB10	< 1

VB11	< 1

VB12	< 1

VB13	< 1

VB14	68.6 ± 10

VB17	< 1

To determine the frequency of individual Vb subfamilies expressed by tetramer^+ ^T cells in DR1 mice, draining inguinal cells from DR1 mice immunized 10 days earlier with A12/CFA were incubated with tetramer labeled with PE, anti-CD4-APC, anti-a/bTCR-PerCP, and an anti-TCR-Vβ-specific Ab conjugated with FITC. Labeled cells were analyzed from each sample with flow cytometry, and the final analysis was performed by using FlowJo software. Data shown represent the mean values from three separate analyses. Virtually all the tetramer^+ ^T cells expressed either the Vβ14 or the Vβ8 (Vβ8.1, Vβ8.2, or Vβ8.3) TCR segment, identifying them as the major T-cell population responding to the A12 peptide presented by DR1.

**Figure 3:**
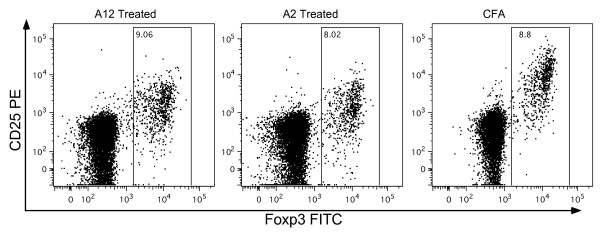
**A12-responsive T cells are not Tregs**. Inguinal lymph node cells from DR1 Tg mice immunized 10 days earlier with A12/CFA (left panel), A2/CFA (middle panel), or Ova/CFA (right panel) were cultured directly *ex vivo *with anti CD25, CD4^+^, VB8^+^, VB14^+^, and intracellular FoxP3. Selective gating was performed on the CD4^+^, VB8^+^VB14^+ ^cells. Before intracellular staining, a sample of the A12-specific cells was stained with DR1-A12 tetramer to confirm that the VB8^+^, VB14^+ ^population was enriched for tetramer^+ ^cells (15% tetramer^+^). The experiments were repeated after 4 days of culture with the A12 peptide *in vitro*, and the results were similar to those shown. These data indicate that the numbers of Foxp3^+^, CD25^+ ^Treg cells remain at background levels after immunization with A12/CFA.

Our next approach was to characterize the phenotype of the heterogeneous CD4^+ ^T-cell subset generated by interaction with A12 by using multiparameter flow cytometry. Naive T cells activated by antigen/APC interaction are expected to expand and differentiate into effector T cells, forming subpopulations that express activation markers, such as CD25, and memory markers, such as CD44. We previously showed that T cells recognizing the immunodominant T-cell determinant of CII upregulate both activation and memory markers on stimulation with CII [7,17]. However, when A12-tetramer^+ ^cells were tested for the presence of activation and memory markers, their expression (CD25 and CD44) was undetectable either directly *ex vivo *or after 4 days of culture (data not shown). Similarly, the numbers of Tregs (identified as Foxp3^+ ^and CD25^+^; Figure 3) remain at background levels in populations of CD4^+^, VB8^+^, and VB14^+ ^T cells that have been immunized with A12/CFA. Taken together, our data reveal that a population of A12-specific inhibitory T cells migrates to the inguinal lymph nodes after immunization with A12/CFA and develops a distinct oligoclonal TCR response without memory, activation, or Treg-specific identifiers.

### Mechanism of suppression

**Figure 4:**
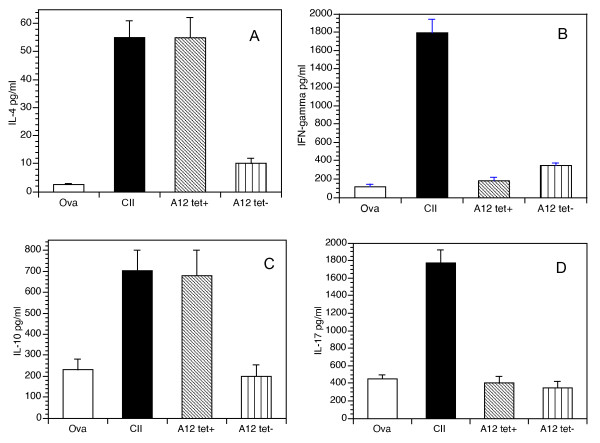
**Cytokine responses to A12-tetramer^+ ^cells**. DR1 mice were immunized with Ova (open bars), CII (solid bars), or A12 (hashed and striped bars), and the draining inguinal lymph node cells were selected to obtain the CD4^+ ^populations. The A12-immunized lymph node cells were further fractionated into tetramer^+ ^(left hashed bars) and tetramer^- ^(striped bars) populations. The cells were cultured (5 × 10^5 ^CD4^+ ^T cells/ml) with wild-type APC (DR1-positive splenocytes; 1:2 ratio) prepulsed *in vitro *with the immunodominant collagen peptide. Supernatants were collected and tested for the presence of each cytokine by using a multiplexed ELISA. The supernatants were collected from Ova-specific CD4^+ ^T cells (open bars), CII-specific T cells (solid bars), DR1-A12 tetramer^+ ^T cells (left hashed bars), or tetramer^- ^T cells (striped bars). Results shown represent the mean ± SD of three separate experiments and are expressed in picograms per milliliter of culture supernatants. The Th1/Th2 ratio was 13:1 in T cells responding to CII, whereas the Th1/Th2 ration was 0.5:1 in the DR1-A12 tetramer^+ ^T cells.

A major mechanism by which T cells function is through the secretion of cytokines, either inflammatory (Th1, Th17) or suppressive (Th2). To test for cytokine secretion, cells from the inguinal lymph nodes of mice previously immunized with A12/CFA were fractionated with ferromagnetic beads into tetramer^+ ^and tetramer^- ^subsets and cultured with APCs pulsed with the immunodominant collagen peptide. Control T cells were purified from mice immunized with either CII or Ova. As shown in Figure 4, supernatants from tetramer^+ ^cell cultures contained predominantly Th2/suppressive cytokines (IL-4 and IL-10) in response to collagen, whereas the tetramer-negative population did not react. Conversely, control A2-specific T cells (from CII-immunized mice) secreted both suppressive (IL-4 and IL-10) and inflammatory (IFN-γ and IL-17) cytokines to collagen, whereas Ova-responsive cells were generally nonreactive. These data make it clear that tetramer^+ ^cells are responsible for the suppressive cytokine secretion after activation by A12. Very little evidence suggests that Tregs, NKT cells, or any other potential suppressive subsets play a major role.

To date, our studies of A12-tetramer^+ ^T cells have focused primarily on T cells detected in inguinal lymph nodes after immunization with A12/CFA. Interestingly, when T cells from inguinal lymph nodes of cells immunized with CII/CFA (without treatment with analog) were collected 10 days after immunization and stained for the presence of A12-tetramer^+ ^T cells, we detected a small population (0.86 ± 0.2 A12-tetramer^+ ^T cells compared with 0.23 ± 0.04 tetramer^+ ^T cells from Ova immunized mice (*P *≤ 0.007 from three separate analyses). Although more work must be done to characterize definitively the cytokine profiles and phenotypes of these cells, we have found that T cells collected from lymph nodes after immunization with CII/CFA secrete suppressive cytokines (primarily IL-4 and IL-10) in response to A12/DR1 and not inflammatory cytokines [6]. These data suggest that activated T cells can be redirected to respond to the analog with a suppressive cytokine profile and that this redirection can occur in a setting of active inflammation. Moreover, we have found that the cytokine response to the analog A12 remains suppressive, regardless of which routes were used for administration or whether the peptide was given before or after immunization [6].

### Role of IL-4 and IL-10 in A12 suppression

**Figure 5:**
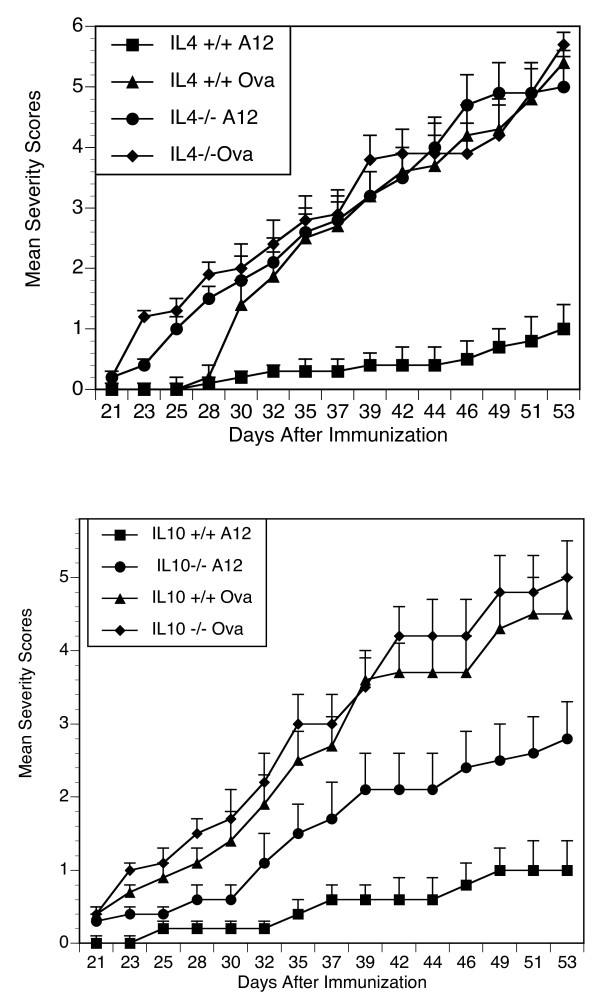
**Analog peptides in IL4^-/- ^and IL10^-/- ^mice**. Groups of DR1^+ ^IL-4^-/- ^, DR1 IL4^+/+ ^littermates, DR1 IL10^-/-^, and DR1 IL10^+/+ ^littermate mice were immunized with CII/CFA. Mice were treated with either the A12 peptide or Ova (0.6 mg) subcutaneously in IFA at the time of immunization. The mean severity scores for arthritis are shown at various times after immunization. The DR1 IL4^+/+ ^mice treated with A12 differ significantly from DR1 IL4^+/+ ^mice treated with Ova (*P *< 0.0005 on day 53). Conversely, the DR1 IL4^-/- ^group treated with A12 did not differ from Ova-treated controls. Data shown are representative of four separate experiments. Similarly, the DR1 IL10^+/+ ^mice treated with A12 were significantly suppressed when compared with the DR1 IL10^+/+ ^mice treated with Ova, *P *< 0.001 on day 53). Although the DR1 IL10^-/- ^mice had suppression of arthritis compared with Ova-treated controls (*P *≤ 0.05), the suppression was not as potent as that obtained with DR1 IL10^+/+ ^mice. Similarly, the incidence of arthritis was 15% in IL10^+/+ ^A12-treated mice, which was significantly different from that of OVA-treated IL10^+/+ ^mice (67%; *P *= 0.004). Conversely, the A12-treated IL10^-/- ^mice had an arthritis incidence of 36%, which was not statistically different from the IL10^+/+ ^Ova-treated mice. Data shown are representative of three separate experiments.

We previously showed that IL-4 can attenuate arthritis. However, it remained unclear whether other cytokines affect the A12 mechanism of action. To confirm and compare the importance of other Th2 cytokines *in vivo*, we developed DR1 mice that were genetically deficient in IL-4 and IL-10, so that groups of 10 IL-4^-/- ^or IL-10^-/- ^mice could be treated with A12 at the time of immunization with CII/CFA and compared with wild-type DR1 mice for the severity of the development of arthritis. As shown in Figure 5, the cytokine IL-10 appears to play a partial role in the A12 prevention of arthritis (Figure 5), whereas IL-4 is more potent. Interestingly, T cells from A12-treated IL-10^-/- ^mice produced IL-4 when cultured with collagen (IL-4 = 76 ± 21 pg/ml), but the levels were not different from those produced by A12-treated IL-10^+/+ ^T cells (IL-4 = 65 ± 14 pg/ml). These data extend our previous observation to confirm that the effectiveness of A12 in treating arthritis is mediated at least in part by both IL-4 and IL-10.

#### Treatment of arthritis

**Figure 6:**
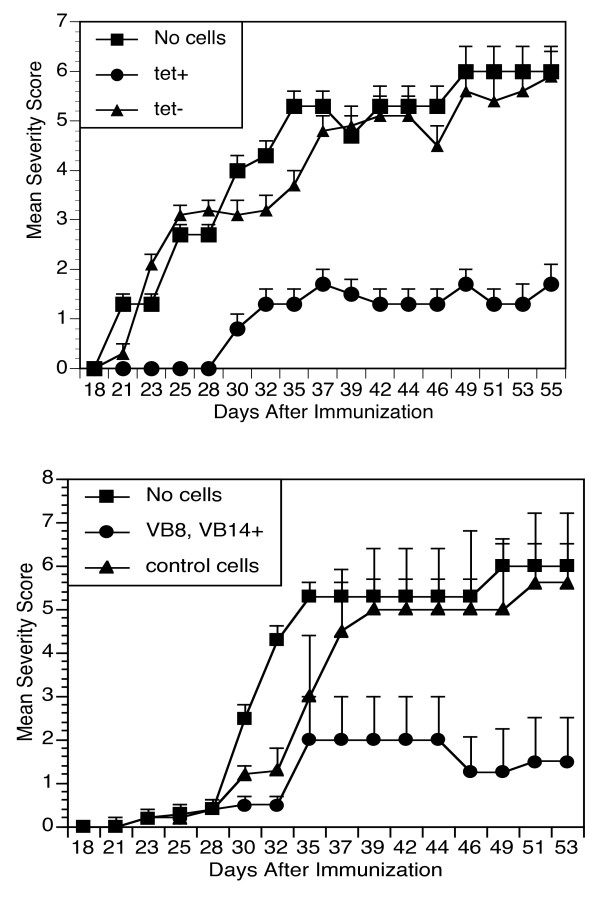
**Cell transfer: prevention of arthritis**. Tetramer^+ ^T cells from draining lymph nodes were collected from DR1Tg mice immunized 10 days earlier with A12/CFA. DR1 mice (*n *= 3) were infused with 2 × 10^5 ^cells of either tetramer^+ ^or tetramer^-^, A12-primed CD4^+ ^T cells. All recipients were immunized with CII/CFA on the day of the cell transfer and observed for arthritis. **(A) **Mean severity scores over time. As indicated, only the tetramer^+ ^A12-immune cells could prevent collagen-induced arthritis (final severity scores comparing square versus triangle of 1.3 ± 2.5 versus 5.4 ± 3.2; *P *≤ 0.01. The cell-transfer experiments have been repeated with both unfractionated cells and VB8^+^, VB14^+ ^purified T cells from A12-immunized lymph nodes with similar results. Suppression of established arthritis **(B)**. Inguinal lymph node cells from A12-immunized DR1 mice were fractionated into a VB8, VB14^+ ^population and a VB8, VB14^- ^population. DR1 mice (*n *= 10) were immunized with CII/CFA and observed for the development of arthritis. On day 23 after immunization, when arthritis was established, each mouse was infused intravenously with 5 × 10^5 ^cells of either VB8, VB14^+ ^T cells (enriched for 7.5 × 10^4 ^tetramer^+ ^cells) or VB8, VB14^- ^T cells. (B) Mean severity scores over time. As shown, only the VB8, VB14^+ ^A12-immune T cells could suppress active arthritis in the collagen-induced arthritis model (final severity scores comparing (triangle versus circle) of 1.5 ± 1.0 versus 5.6 ± 1.6; *P *≤ 0.03). Data shown are representative of three separate experiments.

To resolve whether tetramer-positive T cells could successfully suppress arthritis in the CIA model, we performed passive transfer experiments [18]. Draining inguinal lymph node cells from DR1/TCR Tg mice previously immunized with A12/CFA were collected, and tetramer^+ ^cells were fractionated by using ferromagnetic beads. Either tetramer^+ ^cells or tetramer^- ^controls were infused intravenously into DR1 tg mice, by using a (prevention protocol) so that mice could be observed for effects on collagen-induced arthritis. When infused before the induction of arthritis, we observed (Figure 6A), that the tetramer^+ ^population of A12-immune T cells was extremely effective in suppressing arthritis, compared with tetramer-negative control LN cells.

With a different strategy to enrich for A12-responsive T cells, we collected Vβ8 and Vβ14 populations from A12-immunized lymph nodes by means of ferromagnetic beads and subset-specific antibodies for positive selection. Flow cytometry confirmed that this population was highly enriched for the tetramer^+ ^T cells before infusion into mice. When these cells were given to mice after arthritis had been established (Figure 6B), arthritis was significantly attenuated. Mean antibody titers to CII taken 6 weeks after immunization were decreased in mice given the Vβ8^-^, Vβ14^- ^enriched T cells, compared with mice given Vβ8, Vβ14^- ^CD4^+ ^T cells (Table 2).

**Table 2:** Cytokine responses to murine CII in mice treated with A12-immune T cells

Treatment	Cytokines	(pg/ml)		Antibodies to CII
	**IFN-γ**	**IL-17**	**IL-4**	

VB8^+^, VB14^+ ^T cells	283 ± 25 (*P *≤ 0.05)	2,826 ± 320 (*P *≤ 0.03)	29 ± 5 (*P *< 0.05)	29 ± 3 (*P *≤ 0.05)

VB8^-^, VB14^- ^T cells	2,570 ± 120	10,856 ± 75	10 ± 2	77 ± 30

Inguinal lymph node cells from A12-immunized DR1 mice were fractionated into a Vβ8, Vβ14^+ ^population and a Vβ8, Vβ14^- ^population by using ferromagnetic beads. DR1 mice (*n *= 10 for each group) were immunized with CII/CFA, and on day 23 after immunization, when arthritis was established, each mouse was infused intravenously with 5 × 10^5 ^cells of either Vβ8, Vβ14^+ ^T cells or Vβ8, Vβ14^- ^T cells. To confirm the importance of the cytokine responses after cell transfer *in vivo*, inguinal lymph node cells were taken from the mice 28 days after treatment and were cultured with murine collagen so that supernatants could be analyzed for the presence of cytokines. When cultures from mice treated with the Vβ8^+^, Vβ14^+ ^cells were compared with those from mice treated with the Vβ8^-^, Vβ14^- ^cells, the IL-4 response to murine collagen was elevated, whereas the inflammatory cytokines (IFN-γ and IL-17), were suppressed. Mean antibody titers to CII in sera taken 6 weeks after immunization were similarly suppressed in mice given the Vβ8, Vβ14^+^, enriched T cells, compared with mice given Vβ8, Vβ14^- ^CD4^+ ^T cells.

To confirm the effectiveness of tetramer^+ ^T cells *in vivo*, inguinal lymph node cells were taken from the mice 28 days after treatment and were cultured with collagen so that supernatants could be analyzed. When cultures from mice treated with the Vβ8^+^, Vβ14^+ ^cells were tested for cytokines, the IL-4 response to murine collagen was increased, whereas the levels of the inflammatory cytokines were decreased when compared with those from mice treated with the Vβ8^-^, Vβ14^- ^cells (Table 2). Collectively, the *in vivo *and *in vitro *experiments confirm that the suppression of arthritis by A12-specific T cells involves active secretion of suppressive cytokines (specifically IL-4 and IL-10), resulting in a downregulation in the inflammatory cytokines IL-17 and IFN-γ.

## Discussion

The development of class II MHC tetramers has been more challenging than that of class I tetramers, in part because, unlike class I (which can be assembled from HLA heavy chain, and excess peptide and β_2_-microglobulin), the class II molecule is an assembly of α and β chains, both of which are required for peptide binding. Proper folding is required for the formation of a stable complex. The most successful strategy involves producing empty class II molecules that can be loaded with exogenous peptide and assembled as MHC tetramers. However, A12 has an extremely low binding avidity for the MHC, making that approach unlikely to be successful. In this study, we developed a highly specific HLA-DR1-A12 tetramer in which we overcame the low-avidity problem by covalently linking the peptide to the DR1. This strategy allows successful identification of A12-specific T cells in the wild-type mouse.

*Ex vivo *studies revealed the expansion of A12-specific T cells in the inguinal lymph nodes after treatment with the peptide. Moreover, we showed that DR1-A12 tetramer^+ ^T cells have significant antigen-specific characteristics, including a limited TCR repertoire, and the expression of high levels of Th2/suppressive cytokines despite constituting only 1.5% or less of the CD4^+ ^population *in vivo*. The population of A12-specific T cells can be expanded *in vitro *to increase fivefold after 4 days of culture. Although the numbers of A12-specific T cells may be small in terms of the total CD4^+ ^T-cell population, this still represents at least a 1,000-fold expansion of these cells, given an estimated antigen-specific T-cell precursor frequency of 10^5 ^to 10^6^. In all, this approach demonstrates that a detailed *ex vivo *image of the developing T-cell response to an analog peptide can be achieved by using class II tetramers, and our data suggest that analog-specific T cells can expand *in vivo *and remain detectable in draining lymph nodes for a significant period.

The T cells identified by the A12-CII tetramer expressed primarily Th2/suppressive cytokines (IL-4 and IL-10) that are associated with autoimmune suppression. In comparison, control cells specific for collagen secreted significant amounts of all subclasses of cytokines, both inflammatory (IFN-γ, IL-17) and noninflammatory (IL-4 and IL-10). Both IL-10 and IL-4 are known to suppress CIA when they are administered to mice [19-22]. By using mice genetically deficient in either IL-4 or IL-10 that have been treated with the A12 peptide, we now extend our previous studies to demonstrate that both cytokines play an important role in the function of A12, IL-4 > IL-10. Our previous work is consistent with the concept that Th2/suppressive cytokines have a unique role in the suppression of arthritis [23]. These elevated levels of Th2 cytokines in the tetramer^+ ^T cells support their identification as A12-specific T cells. They probably play a significant role in promoting the development of suppression in this arthritis model.

This tetramer-based approach to the analysis of an autoimmune T-cell response offers several advantages over conventional approaches. First, cells are studied *ex vivo*, thus avoiding any tissue-culture effects on gene expression and T-cell function. Stable tetramer binding to T cells can be achieved within 2 hours, and the assay is performed at physiologic temperatures. Second, all measurements of phenotype and function are focused on T cells of the specificity of interest instead of an averaged analysis of the total T population derived from the lymphoid tissue. Although the frequency of the Ag-specific CD4^+ ^T cells of interest is low both in our analyses and in those of others [24], this problem can be overcome by analysis of large numbers of cells and by focusing the analysis on specific subpopulations. In terms of data acquisition and analysis, we found that care must be taken in quantitating these low-frequency events, because background or nonspecific fluorescence can significantly compete with a 1% or less positive population. The use of both inclusion (CD3^+ ^or CD4^+^) and exclusion (CD8^-^/CD19^-^) gates for data analysis helped to ensure the high degree of sensitivity and reproducibility in identifying these low-percentage events.

Although the A12-specific T-cell response could be clearly visualized by our DR-A12 tetramers, some questions remain as to whether these tetramers detect all the DR1-restricted CD4^+ ^T cells specific for the A12 peptide. Although the frequency of A12-specific T cells we observed is similar to that reported in other MHC class II tetramer-based studies of Ag-specific T cells, it is still possible that we are failing to identify some small population of CII-specific T cells that might have low affinity for DR1-A12 or for some other unknown reason fail to bind the tetramer. Despite these concerns, however, this tetramer-based approach has significantly broadened our understanding of the role and function of analog-specific T cells in wild-type mice. The detection of A12-specific T cells in the inguinal lymph nodes of arthritic animals after treatment with peptide and the discovery that tetramer^+ ^T cells can suppress arthritis when infused into arthritic mice indicates that these cells may play a direct role in altering the course of autoimmune disease.

## Conclusions

The success of the altered peptide ligand, glatiramer acetate (Copaxone), in treating patients with multiple sclerosis (MS), suggests that peptide therapies based on naturally occurring proteins may provide an effective alternative for the treatment of autoimmune arthritis. Copaxone is a mixture of peptides composed of four randomly arranged amino acids (L-alanine, L-lysine, L-glutamic acid, and L-tyrosine), whose chemical structure resembles that of the myelin basic protein molecule [25]. The mechanism of action of successful APLs appears to be the induction of inhibitory T cells, so that inactivated T cells become Th2/inhibitory cells (which secrete antiinflammatory cytokines) instead of Th1 and Th17 cells (which secrete proinflammatory cytokines), a phenomenon known as "immune deviation." The effectiveness of A12 is based on its ability to transform both potential inflammatory T cells and/or bystander T cells into therapeutic (regulatory-like) T cells [3]. Moreover, this deviation also downregulates other proinflammatory Th1 and Th17 cells, regardless of their antigenic specificity, in a process known as "bystander suppression." APLs are potentially safer than existing therapies because they contain a modification of an endogenous naturally occurring protein, used to arrest the autoimmune T-cell attack and allow tissue repair.

We demonstrated that peptide/MHC multimers can be used to detect inhibitory cells induced by altered peptide ligands, even when the peptides have extremely low avidity for the MHC. These cells are highly effective in suppressing autoimmune arthritis. These data demonstrate that analog-specific multimers are powerful tools for detecting inhibitory T cells, useful for studies of human T cells in upcoming clinical trials for RA. We anticipate that future studies will outline the differences between A12-specific T cells from healthy control subjects and patients with RA.

## Abbreviations

A2: a peptide containing the immunodominant determinant sequence of CII (GIAGFKGEQGPKGEB); A12: a synthetic peptide representing the sequence (GIAGNKGDQGPKGEB); APC: antigen-presenting cell; APL: altered peptide ligand; B: hydroxyproline; CIA: collagen-induced arthritis; CII: type II collagen; MHC: major histocompatibility complex; RA: rheumatoid arthritis; TCR: T-cell receptor; tg: transgene.

## Competing interests

The authors declare that they have no competing interests.

## Authors' contributions

All authors made substantial contributions to the conception and design of the experiments. MK and DLC designed and carried out all the studies analyzing the T cells *in vitro*; EFR designed and developed the tetramers; MLB and DDB designed and participated in the studies using flow cytometry; LKM, JS, and AHK designed and participated in studies using the collagen-induced arthritis animal model. All authors were involved in drafting and revising the manuscript critically for important intellectual content. All authors read and approved the final manuscript.
